# Supplementary figures and images for: Macrophage-specific responses to human- and animal-adapted tubercle bacilli reveal pathogen and host factors driving multinucleated cell formation
Source: PLoS Pathog. 2021 Mar 15;17(3):e1009410. doi: 10.1371/journal.ppat.1009410 (PMC7993774; doi:10.1371/journal.ppat.1009410)

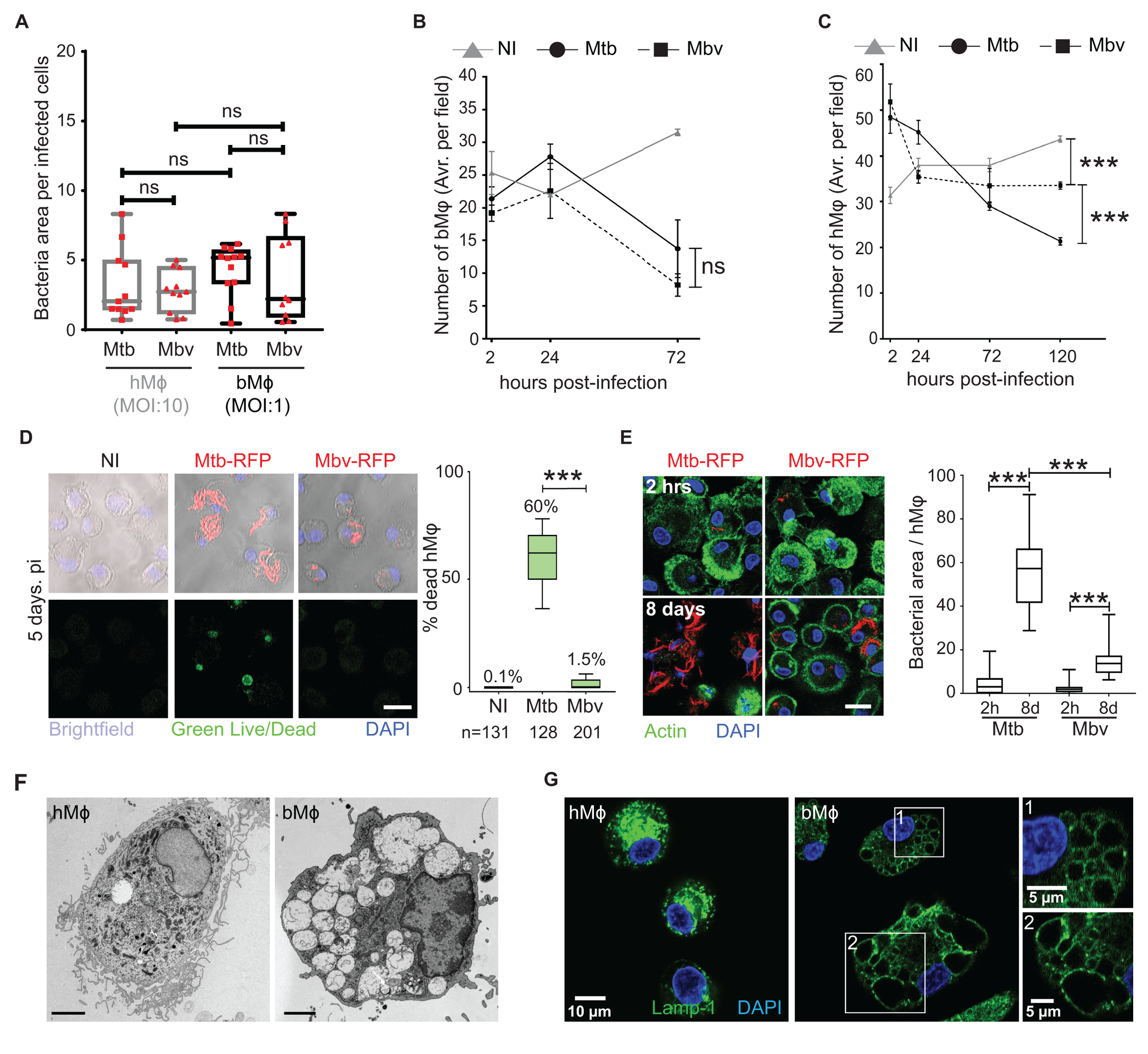

Supplement: S1 Fig — (A) hMϕ were infected with Mtb-RFP or Mbv-RFP at an MOI of 10, whereas bMϕ were infected at an MOI of 1. Intracellular bacteria were quantified based on intracellular RFP signal and expressed in bacteria area per infected cells. Each dot represents the average of 10 fields from 2 independent experiments (also with different donors). (B) Evolution of the number of bMϕ during the course of infection (2 to 72 hours post-infection). (C) Evolution of the number of hMϕ during the course of infection (2 to 120 hours post-infection). (D) Left panel: Confocal images of hMϕ infected with Mtb-RFP or Mbv-RFP after 5 days of infection. Brightfield was used to visualize the cells. Bacteria are visualized in red, cell nuclei were stained with DAPI (blue) and nuclei from dead cells in green. Scale bar: 20 μm. Right panel: Quantification of the level of cytotoxicity based on Green Live/Dead stain; uninfected. Non-infected cells (NI) were used as a control, n represents the number of cells analysed. (E) Left panel: Confocal images of hMϕ infected with Mtb-RFP or Mbv-RFP for 2 hours or 8 days. Actin (in green) was used to visualize the cells. Cell nuclei were stained with DAPI (blue) and bacteria-RFP are visualized in red. Scale bar: 20 μm. Right panel: quantification of intracellular growth expressed in bacteria area (μm2) per hMϕ. Data are representative of 2 independent experiments. (F and G) hMϕ and bMϕ after 7 days differentiation with GM-CSF. (F) Representative electron microscopy images of uninfected hMϕ and bMϕ. Scale bar: 5 μm (G) Representative confocal images of uninfected hMϕ or bMϕ fluorescently stained for the late endosomal marker Lamp-1. The regions in the white squares are highlighted on the right-hand side of the micrograph. (TIF) [file ppat.1009410.s001.tif]

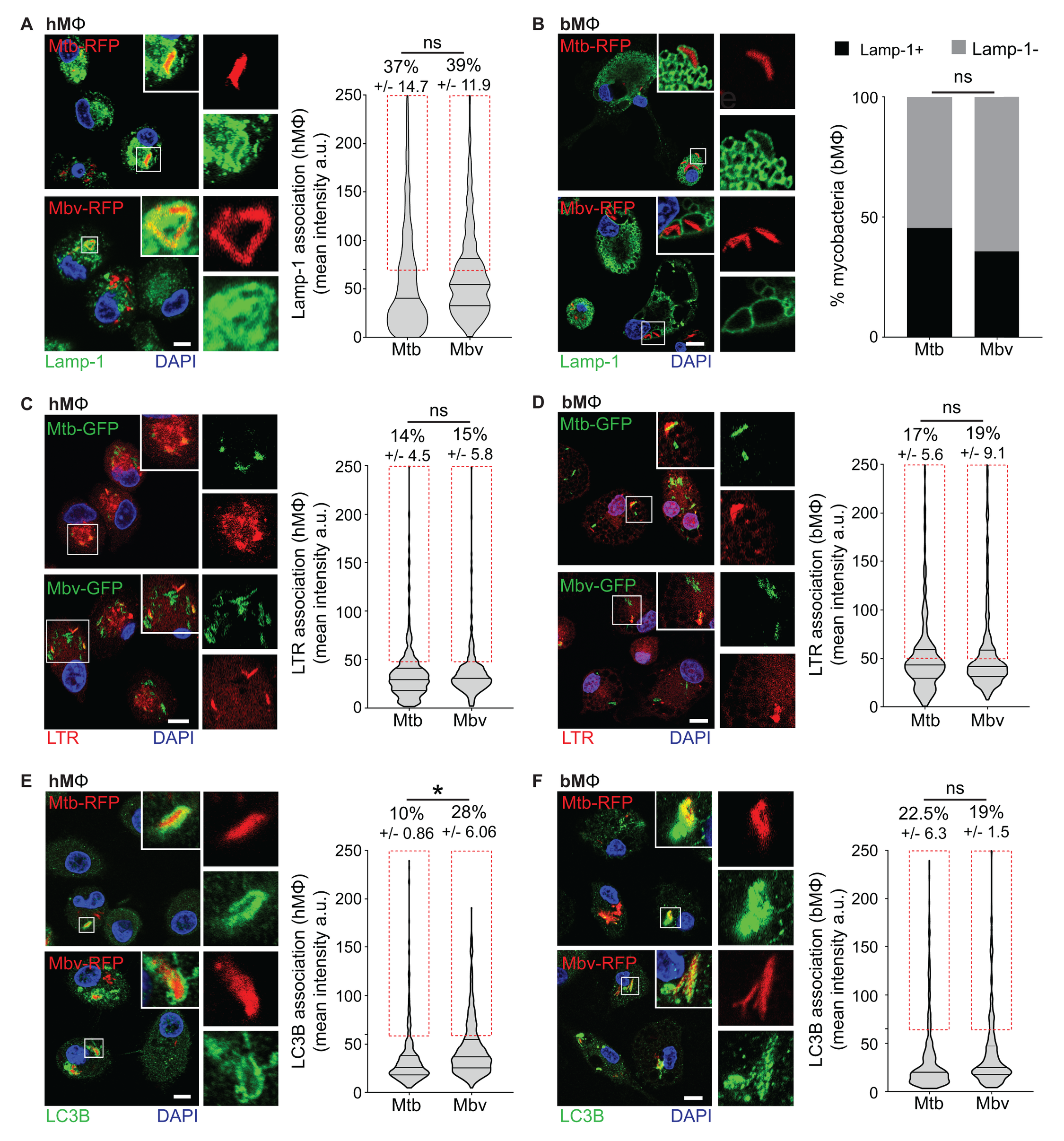

Supplement: S2 Fig — hMϕ or bMϕ infected with Mtb-RFP or Mbv-RFP (A, B, E and F) or Mtb-GFP or Mbv-GFP (C and D) for 24 h. Samples were fixed and fluorescently stained for the late endosomal marker Lamp-1 (A and B), for the pH sensitive dye LysoTracker DN99 Red (LTR) (C and D) and for the autophagic marker LC3B (E and F). For each fluorescent confocal image, the cell nuclei were stained with DAPI. Positive association of bacteria with the different markers, delimited by a white square, are magnified and displayed at the top right corner and the right-hand side of each image. Scale bars represent 10 μm. Graphs represent the quantification of the marker association with Mtb or Mbv ± SEM from three independent experiments. Each dot represents the mean relative fluorescent intensity of the cellular marker with a single or distinct bacteria group. The population within each dotted red box corresponds to the percentage (± STD) of bacteria positive for the marker tested. (TIF) [file ppat.1009410.s002.tif]

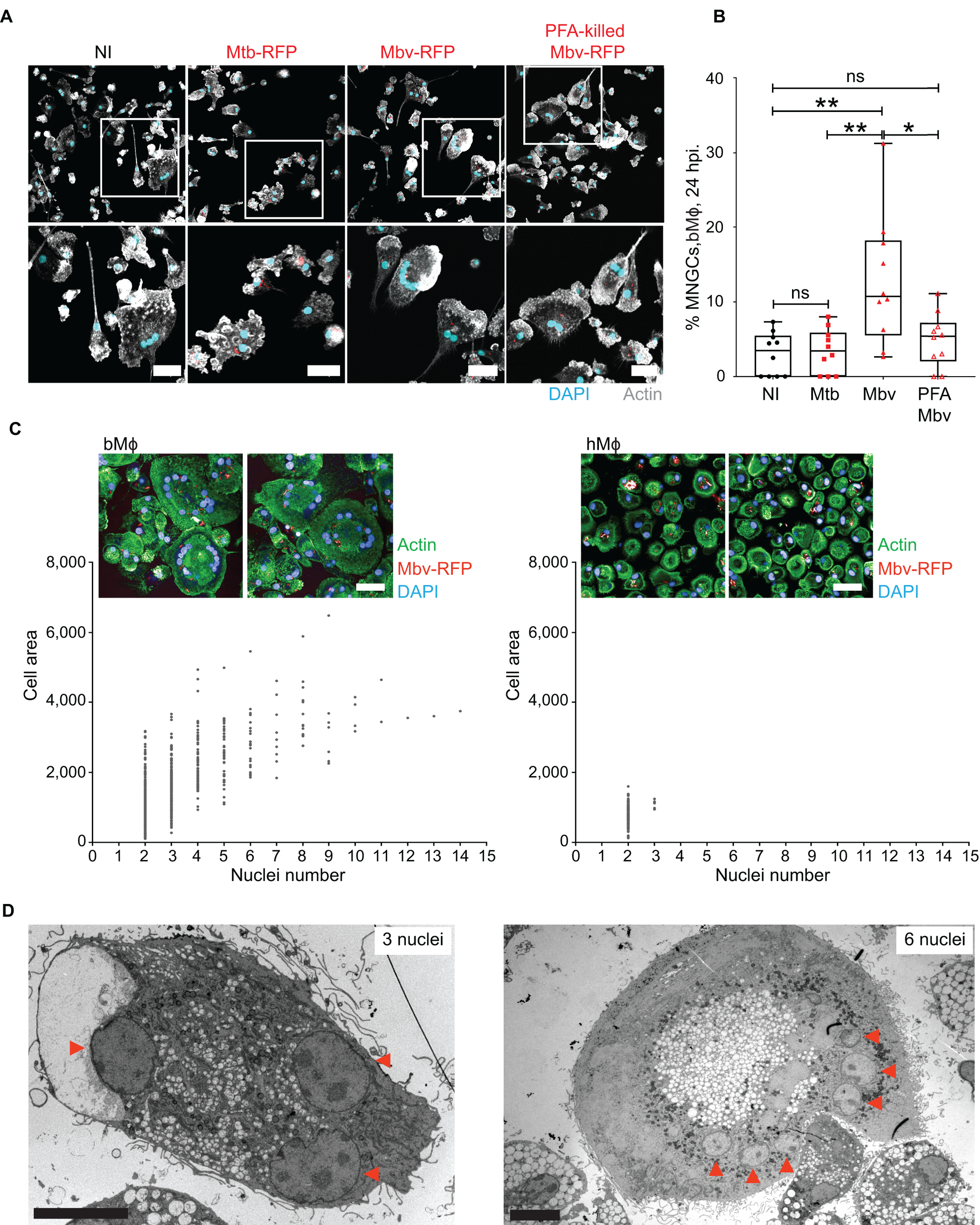

Supplement: S3 Fig — (A) Fluorescence confocal images of bMϕ infected with Mtb-RFP, Mbv-RFP or PFA-killed-Mbv-RFP for 24 h. Non-infected cells (NI) were used as a control. The bacteria are visualized in red, the cells-actin cytoskeleton is in white (phalloidin-488) and cell nuclei (DAPI) in cyan. The white square represents a region of interest magnified below each image. Scale bar, 40 μm. (B) Quantification of the percentage of MNGCs in bMϕ for each condition. Data are representative of two independent biological repeats, each carried out in duplicate. (C) GM-CSF-bMϕ or -hMϕ were infected with Mbv-RFP (red) for 24 h. PFA-fixed infected cells were stained with phalloidin-Alexa Fluor 488 (actin, green). DAPI (blue) was used to stain nuclei. Images presented above were acquired using a confocal microscope. Images were analysed using Harmony software (PerkinElmer). Actin stain was used to mask the cell bodies and determine the number and the area of the cells detected. DAPI staining was used to segment and count the number of nuclei in each cell. Cells containing 2 or more nuclei were considered. For each cell represented by a grey dot, values were plotted as nuclei number as a function of the cell’s area (μm2). Scale bar, 50 μm (D) Electron microscopy image of Mbv-induced bovine MNGCs containing three or six distinct nuclei (red arrows). Scale bar, 10 μm. (TIF) [file ppat.1009410.s003.tif]

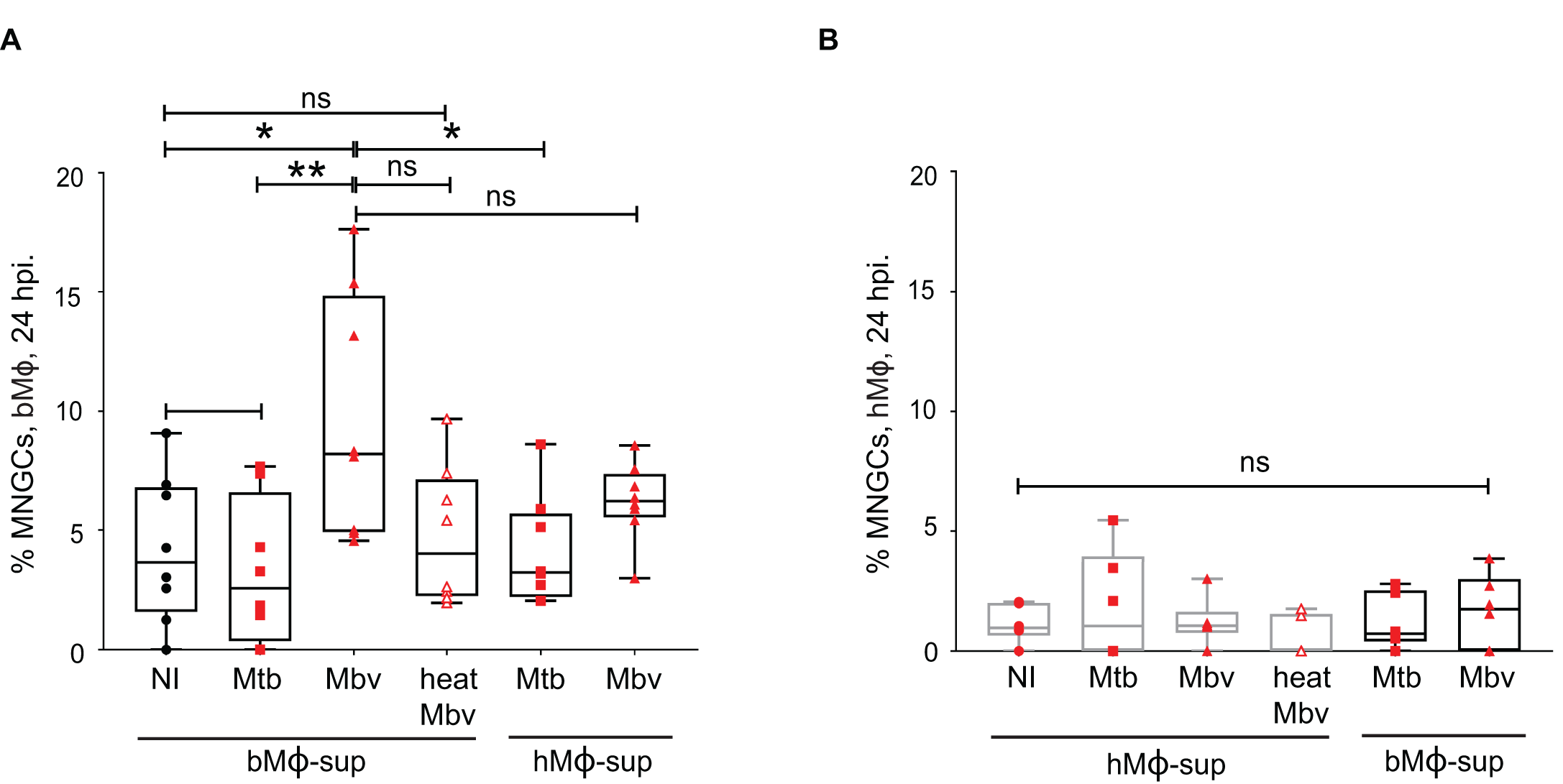

Supplement: S4 Fig — (A) Supernatant transfer assay from bMϕ (non-infected (NI), infected with Mtb or Mbv), or from hMϕ infected with Mtb or Mbv for 24 h, onto naïve bMϕ. A fraction of supernatant from bMϕ-infected with Mbv was heat inactivated and used to stimulate naïve bMϕ (heat Mbv). Graph represents the number of MNGCs formed in cultures of naïve bMϕ following the addition of 400 μl of supernatant derived from cultures of bMϕ infected with Mbv or Mtb (B) Supernatant transfer assay from non-infected hMϕ (NI), hMϕ-infected with Mtb, Mbv, or bMϕ infected with Mtb or Mbv for 24 h, onto naïve hMϕ. A fraction of supernatant from hMϕ-infected with Mbv was heat inactivated and used to stimulate naïve hMϕ (heat Mbv). The graph represents the quantification of MNGCs for each condition tested. (A and B) Data are representative of two independent experiments, each carried out in duplicates. (TIF) [file ppat.1009410.s004.tif]

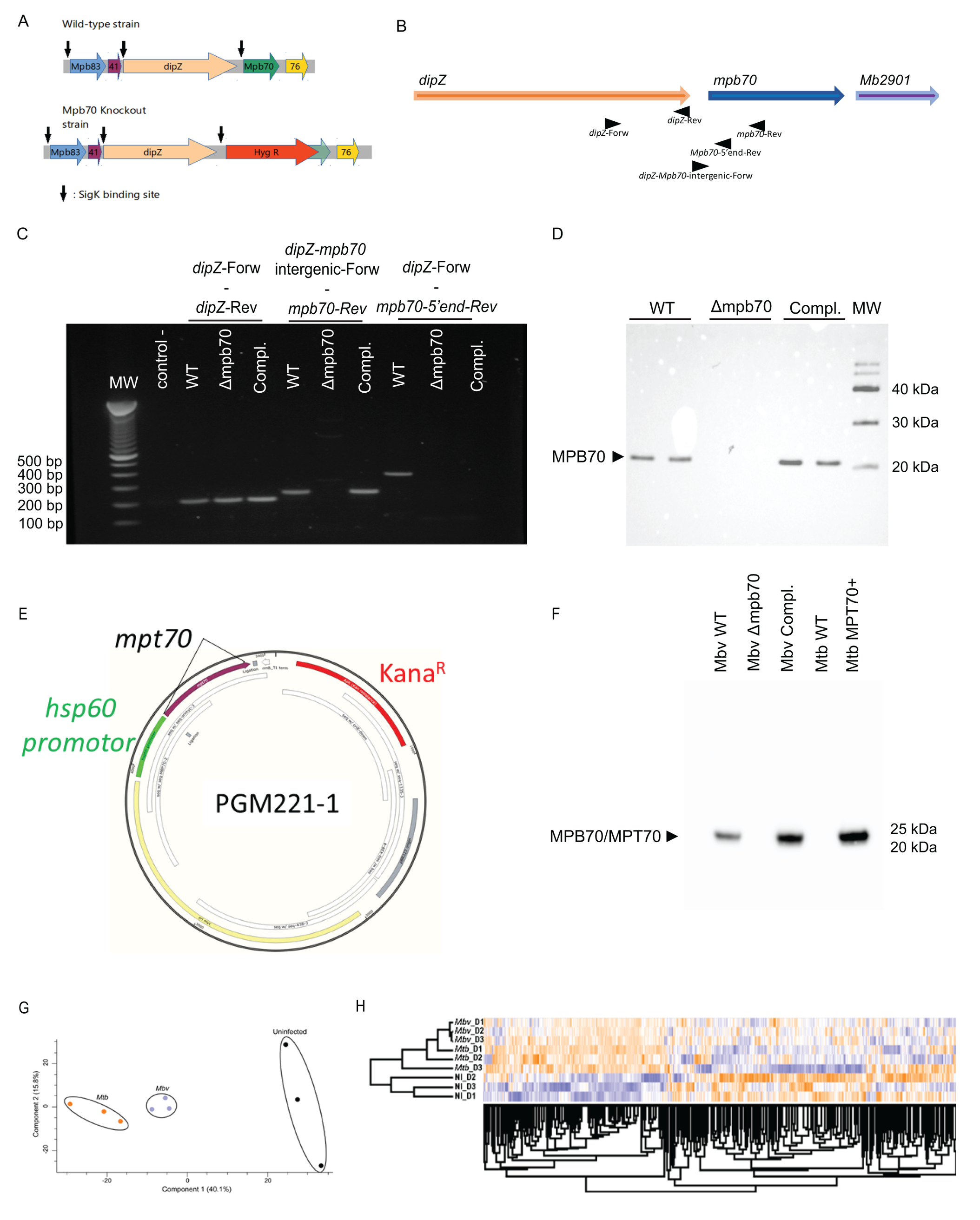

Supplement: S5 Fig — (A) Genetic arrangement Mbv wild-type (WT) and Mbv mpb70 knock-out (Δmpb70) strains. (B) Genetic organisation of dipZ-mpb70 genes and primer locations (C) Characterisation of Mbv mutants by PCR. Deletion of mpb70 and maintenance of dipZ in Mbv Δmpb70 and complemented compared to WT. dipZ is amplified in all the strains. mpb70 (244 bp) was amplified in the WT strain but not in Δmpb70 and complemented strains. The PCR with a forward primer in dipZ and reverse primer in mpb70 gave the expected amplification of a 370 bp product for WT, but was absent in Δmpb70. Its absence from the complemented Δmpb70/mpb70 (Compl) strain, confirms that the location of the mpb70 gene, carried by the replicative plasmid pEW70c2, is distal to the wild type chromosomal location. (D) Western immunoblot for detection of MPB70 in the supernatant of the Mbv WT, Δmpb70 and complemented (Mbv-Compl) strains. Each sample was analysed in duplicate. A 23 kDa band corresponding to MPB70 was detected for the WT and complemented strain but not for Δmpb70 strain. (E) Graphical map of the plasmid pGM221-1 used to generate the strain Mtb overexpressing MPT70 (Mtb-MPT70+). The Mtb-H37Rv gene mpt70 is expressed under the control of hsp60 promotor. The clonality of the Mtb-MPT70+ strain was sustained by the Kanamycin resistance cassette carried by the plasmid pGM221-1. (F) Western immunoblot for detection of MPB70 and its homologous MPT70 in the supernatant of the Mbv WT, Δmpb70, Mbv-Compl, Mtb WT and Mtb-MPT70+ strains. A 23 kDa band corresponding to MPB70 was detected for Mbv WT, Mbv-Compl and MTB-MPT70+ strains but not for Mbv Δmpb70 and Mtb WT strains. (G) Principal component analysis of secretome samples. Distinct clusters of sample groups are observed that indicate high reproducibility between replicates and major protein expression differences between the samples analysed. (H) Unsupervised hierarchical clustering of secretome samples. Distinct clusters of sample groups indicate high reproducibility of [file ppat.1009410.s005.tif]
