# Supplementary material for: Macrophage-specific responses to human- and animal-adapted tubercle bacilli reveal pathogen and host factors driving multinucleated cell formation
Source: PLoS Pathog. 2021 Mar 15;17(3):e1009410. doi: 10.1371/journal.ppat.1009410 (PMC7993774; doi:10.1371/journal.ppat.1009410)
Supplement: S3 Table — (DOCX) [file ppat.1009410.s008.docx]

**S3 Table:** Mycobacterial constructs

| Strains | plasmids | Genetic feature | Selective antibiotic |
| --- | --- | --- | --- |
| H37Rv-RFP | pML2570 | RFP | Hygromycin 50μg/ml |
| *M.bovis* AF2122/97-RFP | pML2570 | RFP | Hygromycin 50μg/ml |
| *M. bovis* wild-type | None | Wild-type | None |
| *M. bovis* ΔMPB70 | phAE159 | *mpb70* deletion | Hygromycin 50μg/ml |
| *M.bovis* ΔMPB70/MPB70  (Mbv-Compl) | phAE159  pEW70c2 | *mpb70* deletion  pEW70c2-mpb70 complementation | Hygromycin 50 μg/ml  Kanamycin 25 μg/ml |
| H37Rv-RFP-MPT70+  (called H37Rv-MPT70+) | pML2570  pGM221-1 | RFP  Constitutive expression of MPT70 (*hsp60* promotor) | Hygromycin 50 μg/ml  Kanamycin 25 μg/ml |
