# Supplementary material for: Macrophage-specific responses to human- and animal-adapted tubercle bacilli reveal pathogen and host factors driving multinucleated cell formation
Source: PLoS Pathog. 2021 Mar 15;17(3):e1009410. doi: 10.1371/journal.ppat.1009410 (PMC7993774; doi:10.1371/journal.ppat.1009410)
Supplement: Attachment — Submitted filename: Point by point_v3_Nov2020.docx [file ppat.1009410.s009.docx]

**Point by point reply**

**Reviewer #1:**

Host tropism is an intriguing feature of tuberculosis, in which almost genetically identical bacilli exhibit very different and specific tropism towards different hosts (i.e. almost exclusively H. sapiens for Mtb, and cattle and several wild animals for M. bovis, with zoonotic cases of human tuberculosis caused by M. bovis). Therefore, understanding the bacterial and host determinants involved in host tropism is indeed an important issue. Here, the authors report on the differential ability of Mtb and Mbov to replicate in human and bovine macrophages and to induce the formation of multinucleated cells (MNCs). They show that while Mtb and Mbov replicate equally in bovine macrophages, Mbov replicates less than Mtb in human macrophages; this is associated with increased access of Mtb to the host cell cytosol in human or bovine macrophages. The most striking conclusion of this study is that only infection of bovine macrophages with Mbov induces the formation of MNCs; this is demonstrated in vitro (Fig 2A, B) and in vivo in experimentally infected cattle (Fig 5). From a mechanistic point of view, the authors show that MPB70 produced by Mbov and extracellular vesicles (EVs) released by Mbov-infected bovine macrophages both contribute to the formation of MNCs. This is an interesting observation on host-pathogen differential interactions due to closely related species of the MTBC.

However, how differential MNC formation explains or is related to host tropism and differential pathogenesis remains uncertain, and the molecular basis for the involvement of MPB70 or EVs in MNC formation remains unexplored. As it stands, the study seems interesting but somewhat descriptive and preliminary. The experiments are convincing and the manuscript is very well written.

We understand some of the concerns of the reviewer. However, we are very surprised this study was found to be preliminary by the reviewer. We report here what is, to our knowledge, the first side-by-side comparison between two macrophage species and two mycobacterial species and from this identified a link with multinucleation that is very specific. We used a mutant in one of the factors and show this factor contributes to this newly described phenomenon. We also validated these observations *in vivo* carrying out experiments with cattle infected with both mycobacterial strains, including histo-pathological verification of the *in vitro* phenoptype. One important aspect the reviewer should consider is the general lack of tools in the bovine system for mechanistic studies. Because the phenotype we report here is only observed in bovine macrophages infected with *M. bovis*, more mechanistic studies on the bovine side are indeed far more difficult. Even the proteomics reported here was challenging because of the lack of accuracy of the *Bos taurus* genome annotation. This lack of tools is also the main reason why there are so few studies on bovine macrophages that investigate host-pathogen interaction with the level of detail and resolution reported here, including imaging and electron microscopy. Instead, most studies are restricting to reporting cytokine secretion and flow cytometry with a very limited panel of antibodies.

Nevertheless, following the suggestions of the reviewer, we have now further characterised the cellular mechanisms operating in MNGCs and show that MNGCs are mostly restrictive for mycobacterial replication (**new Figure 6**). Interestingly, the trafficking of intracellular mycobacteria in MNGCs is significantly different when compared to single nucleated cells and bacteria are localised in acidic compartments, with a higher number of Mbv localised in LC3B positive compartments (autophagosomes, **new Figure 6**).

What is the evidence that multinucleation is “a process that may play a crucial role in MTBC host tropism” (lines 107-108)?

The role of multinucleation in host tropism stated at the end of our introduction originally refers to the specific ability of Mbv to induce the formation of multinucleated cells only in bovine macrophages. Together with the delayed intracellular growth of Mbv in human macrophages, the specific modulation of macrophage responses, observed in our study, supports a differential adaptation of those strains to their respective hosts. We acknowledge that our statement was confusing and we modified it accordingly. The sentence now reads: “Our results identify functional differences between Mbv and Mtb host-pathogen interaction, disclosing a role for MPB70 and EVs in the process of multinucleation in bovine macrophages, and revealing distinct evolutionary adaptations of MTBC to specific hosts.”

From Figure 1B and C, the authors conclude that Mbov and Mtb replicate equally well in bovine macrophages and that Mbov replicates poorly in human macrophages. The use of different Y-axes in the two panels is rather misleading; one might just as well conclude that Mbov replicates about equally well in human and bovine macrophages, but that the growth of Mtb is more limited in bovine macrophages than in human macrophages; in other words, bovine macrophages appear to be more "activated" than their human counterparts, which is consistent with the higher bacterial uptake in bovine cells. This is associated with increased access of Mtb to the cytosol in bovine macrophages (Fig 1F), while Mbov has much greater access to the cytosol in these cells. It could therefore be assumed that Mbov is more adapted than Mtb to the cytosol of bovine macrophages, either because it is more resistant to cytosolic antimicrobial compounds/processes, or because it has better access to or utilization of cytosolic nutrients in bovine cells.

This is indeed an important point, and one that we have discussed as well as the difference between the human and bovine system. Although the cows used in this study are healthy and tested for multiple infections, they are nonetheless reared in the open air and it is possible bovine macrophages are more “activated”. As humans and cattle are biologically dissimilar at multiple levels, it is also not surprising that the two models of human and bovine macrophages present phenotypical differences driven by the species background. For that reason, we preferred to avoid any direct comparisons between both macrophage models but focused on the adaptation of Mtb and Mbv in each different model. From our data, it is clear that *Mbv* growth is strongly delayed in human macrophage compare to Mtb. However, we indeed agree that bMφ might be activated. However, we showed that in both macrophage models, Mtb access to the cytosol is higher than Mbv.

More importantly, how does the MPB70 contribute to the formation of MNCs, and why doesn't MPT70 do so? What explains this difference, and what is the mechanism involved?

MPB70 shares homology with several proteins known to mediate cell-to-cell interaction (Fasciclin 1, Osteoblast-specific factor II or βIgH3). MPB70 may drive bMϕ fusion by mediating cellular interactions and adhesion properties. That is indeed a very interesting question and we believe that the identification of MPB70 in this process will open new areas of research, considering most of the factors identified up to now were related to macrophages and not bacteria. The differences could be mostly related to the levels of expression, known to be different between Mtb and Mbv. Mechanistically, we postulate that high levels of MPB70 could be aid, directly or indirectly, the mechanism of fusion between macrophages. To prove that a mycobacterial protein induced host cell fusion requires extensive experiments and we have tried some of these experiments using recombinant MPB70, but with the main technical problem being that some recombinant proteins induce macrophage activation. Experiments exploring topology and insertion of this mycobacterial protein will be needed (e.g. using artificial membranes) but this is beyond the scope of the current study.

Re. EVs produced by Mbov-infected bovine cells, they contain several potentially interesting candidates, the function of which could have been explored, at least for some of them.

We do agree and appreciate the positive comment regarding our proteomic study. It is true that many of candidates are very interesting. Further analysis would require the validation of specific tools in the bovine system (such as antibodies, siRNA…), which are not currently available. We are interested in further investigate their role in TB pathogenesis in the future. Although we are very enthusiastic to share our findings, we consider that the molecular characterisation of those proteins is beyond the scope of this study.

**Reviewer #2:**

Queval et al. have reported an interesting and original study on the differences between M. tuberculosis (Mtb) and M. bovis (Mbv) in human vs. bovine blood-derived primary macrophages with regards to intracellular survival, location, induction of multinucleated giant cell (MNGC) formation, and the roles of MPB70 and extracellular vesicles in this response. While much work has been done in this area with Mtb and human macrophages, or Mbv and bovine macrophages, this is the first study comparing both mycobacterial species in both human and bovine macrophages. This is very important in understanding the host species specificity of the mycobacterial species. The manuscript is generally well written; the flow of the experiments is smooth and easy to follow. The choice of experiments is logical. The results are clear and generally supported by the figures. The images are generally well presented. My concerns regard the strength of certain statements, the presentation of previous work concerning what is known about macrophages and how MNGCs are formed, and the consistency and clarity of certain terms and figures.

Please replace "MNCs" with "MNGCs" (multinucleated giant cells), as this is what is already used in the literature.

“MNC” had been replaced by “MNGC” in the manuscript and figures.

Please remove the "s" after "MNC" in cases in which it precedes a noun (ie. "MNC formation" or "MNC generation"). Same for "EVs".

This has been modified in the revised manuscript.

"Multinucleation" should not be hyphenated.

This has been modified in the revised manuscript

Some discussion regarding what is already known about factors that promote MNGC formation should be included here (not just in discussion).

As suggested, more information regarding MNCG formation had been added to the introduction:

“In the lung or other organs, the progressive establishment of TB immune response leads to the recruitment of immune cells including CD4+ T cells, CD8+ T cells, as well as both pro- and anti-inflammatory macrophages organized in structure called granulomas. The presence of Multinucleated giant cells (MNGCs; also called Langhans’ cells), containing up to 20 or more nuclei, represents an important feature of granulomatous inflammation [2, 17]. MNGCs are formed from the fusion of pro-inflammatory macrophages recruited at the inflammation cite, and contribute to the global inflammatory response [18, 19]. MNC formation is a multifactorial process involving cytokines such IFN-y, IL-4, TNF-α, IL-2, M-CSF and GM-CSF as well as other fusogenic molecules including carbohydrate, Lipopeptide or secreted cellular factors and receptors including Metalloproteinases, tetraspanins, DC-STAMP and E-cadherin [19-23]. However, none of those fusogenic factors are able to induce MNC by themselves. Although, macrophage differentiation and activation are crucial for MNCs formation, it is still unclear whether the direct interaction of mycobacteria with macrophages promote MNC formation or if it results from the combination of macrophage activation status with the release of specific mycobacterial antigens [24, 25].”

Include your interesting findings that MNGCs are not generally produced by Mbv in hMP or by Mtb.

This is now included in the manuscript:

“We demonstrate that, both in vitro and in vivo, Mbv but not Mtb specifically promotes MNGC formation in bMϕ, arguing that this host-specific MNGC generation contributes to the contrasting pathogenesis between Mtb and Mbv in cattle. Finally, we show that the secreted Mbv protein MPB70 and bMϕ -derived extracellular vesicles (EVs) are bacterial and host factors specifically implicated in bMϕ-multinucleation. Our results identify functional differences between Mbv and Mtb host-pathogen interaction, disclosing a role for MPB70 and EVs in the process of multinucleation in bovine macrophages, and revealing distinct evolutionary adaptations of MTBC to specific hosts.”

Lines 117-119: Please show data.

We apologise for the confusion. The Figure S1A is actually illustrating the point. For more clarity the text has been modified accordingly: “However, bacterial uptake was approximately 10 times higher in bMϕ when compared to hMϕ. In order to normalize the infection protocol, we then infected bMϕ with a lower MOI (MOI of 1) than hMϕ (MOI of 10)”.

Line 190: "Failed" is too strong a word here, since you point out MNGCs in your figure in response to PFA-killed Mbv.

The sentence line 190 now reads: “Multinucleation of bMϕ occurred only with live bacteria since paraformaldehyde-killed-Mbv (PFA-Mbv) presented a number MNGCs similar to Mtb and uninfected control (Fig. S3A and S3B).”

Line 227: Again, "lost its ability" is too strong, as you show MNGCs.

The sentence line 227 now reads: “We found that the deletion of MPB70 markedly impaired the ability of Mbv to induce multinucleation.”

Line 250: Also, "did not induce" has the same problem.

The sentence line 227 now reads: “The percentage of MNGCs significantly increased in bMϕ stimulated with the EVs fraction from Mbv-infected bMϕ, whereas the EVs from Mtb-infected bMϕ did not promote MNCs formation (Fig. 4F and 4G).”

Lines 259-62: Please explain why the cattle were infected with the two mycobacterial species for different lengths of time.

The need for different lengths of time for the cattle experiments was due to time constraints on the INRA large animal containment facilities (Tours, France), requiring a total experimental duration of no more than 16 weeks. Hence animals infected with Mtb H37Rv were maintained for 10 weeks, while Mbv AF2122/97-infected animals were maintained for 6 weeks. Clarification on this point has been briefly added to the “Cattle infection” section under “Cattle infection and histopathological analysis” in the Methods section.

Lines 340-5: You discuss the role of MNGCs in bacterial control, but you state that your MNGCs were devoid of bacteria. Please address.

There is probably some confusion here. We do not understand where in the manuscript we stated that MNGCs were devoid of bacteria. We show in Figure 2, that some MNGCs contain intracellular bacteria. The only statement is that intracellular bacteria are not required to promote bMφ multinucleation.

There should be some discussion of the limitations of your type of macrophages. Blood-derived macrophages are a very different phenotype compared to alveolar macrophages, which are the ones that Mtb and Mbv first encounter in the lung. How relevant or extrapolatable are your findings? What is the phenotype of your particular blood-derived macrophages?

We understand the reviewer’s point and we are aware that the macrophages used here only represent an *in vitro* model. The aim of this work was to identify host and pathogen factors involved in the differing response to Mbv and Mtb in two different macrophage species. Of note, this project started by using alveolar macrophages. However, it was technically difficult to perform experiments with bovine alveolar macrophages because most of the time, and due to the conditions under which cattle are housed, they were contaminated and became activated.

The subset of macrophages involved in TB response is heterogeneous. Macrophages are considered to be one of the main reservoirs for the bacilli, and that their ability to control bacterial proliferation will impact on the outcome of the disease and granuloma formation [1]. GM-CSF macrophages control bacterial burden while M-CSF are considered more permissive to the infection [2, 3]. In the lung, GM-CSF derived alveolar macrophages are essential to control the surfactant homeostasis and the basal lung [4, 5]. Regarding their critical role in lung immunity, GM-CSF would be *per se* considered as the predominant subtype of alveolar macrophages. In our study, we do not claim that our model of GM-CSF derived macrophages behaves as resident alveolar macrophages, nor that resident alveolar macrophages are the only cell subset supporting MNGC formation. Although it is shown that the resident alveolar macrophages provide an early replicative niche for Mtb, the dissemination towards other organs is more likely supported by recruited monocytes including circulating macrophages [6, 7]. And in all the cases there is formation of MNGCs, which demonstrate that MNGCs formation is not only caused by resident alveolar macrophages. These points are now better discussed in our manuscript.

Please present your solutions as "4% PFA-PBS", as this is the conventional presentation.

This has been modified in the revised manuscript.

Please separate numbers from units and "x" from "g" in centrifugation speeds.

This has been modified in the revised manuscript.

Line 778: Please state what test was used to show that your cattle were negative for bovine TB.

The bTB status of this herd was determined by APHA using the standard SICCT method, the tuberculin skin test on the neck. The IFN-gamma release assay is not routinely used in England but can be used in certain circumstances (and more commonly used in Wales). As the freedom of mycobacterial infection was important for this study, the BOVIGAM® IFN Gamma Test was used on all animals. This information has been added to the Methods section, Ethical statements: “Furthermore, all animals were negative for bovine TB using BOVIGAM® IFN Gamma Test, which was conducted in addition to the standard tuberculin skin test.”

Figure 1: What is the number represented in parentheses?

The number in brackets corresponds to the number of bacteria analysed. For more clarity, we have added this information in the legend to Figure 1: “n represents the number of cells analysed. The total number of bacteria analysed corresponds to the number in brackets.”

Figure 2D: Please use arrows to point to the nuclei.

Red arrows pointing the nuclei were added Fig. 2D.

Figure 2E: You state in the results that your bystander MNGCs were uninfected, but your image looks like there is a red dot in the cell.

Bovine macrophage and MNGCs are highly vacuolated, which presents a weak autofluorescence in the red channel. The reddish shade observed in the left MNGCs (stated as uninfected) is due to this weak background, which was intensified by the maximum projection.

Line 1006-7: Please change to "Mbv-infected MNGCs in the bMPh population..." with the exception of the "Ph" (I don't know how to input a symbol in this online reviewing mechanism).

The sentence at line 1006-1007 now reads: “Quantification of the percentage of bystander and Mbv-MNCs in the bMϕ population for two independent donors.”

Figure S1B and C: Please make your symbols as large as they are in Fig. 1B. They are too small to differentiate for people with old eyes like me!

Lines 1063-4: Your hMPh and bMPh graphs are swapped in the figures.

The legend has been modified as follow:

“(B) Evolution of the number of bMϕ during the course of infection (2 to 72 hours post-infection). (C) Evolution of the number of hMϕ during the course of infection (2 to 120 hours post-infection).”

Figure S2: Please label which panels are hMPh and bMPh at the top of the columns for clarity.

hMφ and bMφ labels were added to Figure S2.

Figure S2B: Why is this graph represented differently than all others?

In bMϕ, some bacteria reside in large LAMP-1 positive phagosomes. It is thus not possible to apply a software-based method to determine the mean intensity around those large vacuoles. We thus applied a manual correction to determine the LAMP-1 positive bacteria, which is expressed in our figure as % of mycobacteria in Lamp-1 positive vacuole. This was briefly explained in the results section from line 149 to line 151: “For that, we used two different approaches to quantify the localisation: one for markers closely associated with bacteria (20) and another method for spacious compartments such as the LAMP-1 positive phagosomes (21).” For more clarity, we have added further information in the Methods section, Line 575: “As the marker LAMP-1 was also associated with spacious phagosomes in bMϕ, the same method was used to determine the LAMP-1 positive bacteria with a manual correction for spacious LAMP-1 positive phagosomes. The data were expressed as % of mycobacteria in LAMP-1 positive vacuole.”

Figure S3A: The legend says there are arrows, but I don't see them?

The figure legend had been modified accordingly

Figure S3C: Please use colored arrowheads, as the black ones are hard to see.

The arrows are now red in the revised figure.

Figure S3D, E: Your legend says PFA-killed, but your figures say "heat". Please correct.

We are sorry for this confusion. The paragraph had been modified as follows: “(D) Supernatant transfer assay from bMϕ (non-infected (NI), infected with Mtb or Mbv), or from hMϕ infected with Mtb or Mbv for 24 h, onto naïve bMϕ. A fraction of bMϕ-infected Mbv supernatant was heat inactivated and used to stimulate naïve bMϕ (heat Mbv). Graph represents the number of MNCs formed in cultures of naïve bMϕ following the addition of 400 µl of supernatant derived from cultures of bMϕ infected with Mbv or Mtb (E) Supernatant transfer assay from non-infected hMϕ (NI), hMϕ-infected with Mtb, Mbv, or bMϕ infected with Mtb or Mbv for 24 h, onto naïve hMϕ. A fraction of hMϕ-infected Mbv supernatant was heat inactivated and used to stimulate naïve hMϕ (heat Mbv). The graph represents the quantification of MNCs for each condition tested. (D and E) Data are representative of 2 independent experiments, each carried out in duplicates.”

**Reviewer #3:**

This study reports a comparison of the response of two archetypal host-adapted mycobacterial species, Mycobacterium tuberculosis (Mtb) and Mycobacterium bovis (Mbv) during in vitro interactions with monocyte-derived primary human (hMϕ) or bovine (bMϕ) macrophages. Authors showed that Mtb and Mbv reside in different cellular compartments and differentially replicate in hMϕ, while both strains efficiently replicate in bMϕ. They demonstrated that, both in vitro and in vivo, Mbv specifically promotes multinucleated cells (MNCs) formation in bMϕ, arguing that this host-specific MNCs generation contributes to the contrasting pathogenesis between Mtb and Mbv in cattle. Finally, they showed that the secreted Mbv protein Mtb70 and bMϕ -derived extracellular vesicles (EVs) are bacterial and host factors specifically implicated in bMϕ-multi-nucleation. Because MPB70 is one of the main phenotypic differences between Mbv and Mtb, this protein was an obvious candidate. In the study, one of the strength is having engineered the wild-type strain to abolished MPB70 and found that EV from wild-type but not from deleted bacteria promote MNC. Since MPB70 has homology with the cell adhesion domain Fasciclin 1 (Fas1), Osteoblast-specific factor II (OSFII) or βIgH3, they are suggesting that MPB70 could mediate cell-to-cell contact or cellular adhesion by interacting with cellular receptors such as stabilins, integrins, as well as extracellular matrix components (29, 30). Cell adhesion is a crucial step in the fusion process, bringing Mϕ into close contact with extracellular matrix components. It is tempting to speculate that MPB70 is implicated in bMϕ fusion by mediating cellular interactions and adhesion properties. Their results are very important for the scientific community as they have also identified functional differences between Mbv and Mtb host-pathogen interaction that reveal a role for MPB70 and EVs in the process of multinucleation, a process that may play a crucial role of the mycobacteria tuberculosis complex in host tropism. Overall, the study bring interesting elements but some aspect still require some improvements, notably:

The plasticity of the monocyte-derive macrophages is well known and the impact of the polarization on the innate immune response to pathogen has not been adressed. Serum starvation can induce autophagy. Serum was omitted in the experiments conducted for the observation of extracellular vesicles. It is recommended to test several housekeeping genes and to use the geometric mean the most stable ones for normalization of gene expression. Only one was used for quantification of interferon-β gene (IFNβ). In studies investigating single or double-membrane vacuole, electronic microscopy analysis reveals that LC3 associated pathway involves single-membrane structures whereas macroautophagy is expected to generate double-membrane structures surrounding cargo. This aspect has not been discussed. These are important aspects that need to be discussed in order to guide future investigations.

In the present study, monocytes from blood were differentiated into macrophages for the in vitro experimentations. The plasticity of the monocytes/macrophages is well documented. They can differentiate into either a pro-inflammatory (M1) subtype, also known as a classically activated subtype, or an anti-inflammatory alternatively activated subtype (M2) according to their microenvironment. The plasticity of the monocyte-derive macrophages is well described, which means that the condition of culture influence its differentiation. In this study, GM-CSF was used during differentiation which supports M1 subtype. While the alveolar macrophage is the frontline host immune cell that encounters both M. tuberculosis and M. bovis, is it know what is the predominant subtype of alveolar macrophages?

Please see response to reviewer 2.

The information reported L91-92 (“in each pathogens”) is not exact and might confuse readers. In the study reported by Malone et al (cited ref #11), they have performed detailed comparative transcriptomic analyses of the bovine alveolar macrophage response to infection with both pathogens using RNA-sequencing (RNA-seq). The number of differential expressed genes reported were identified in bovine alveolar macrophages in response to Mtb or Mbv infection and not in each pathogens. As reported in Malone et al 2018, the number of DE genes and proteins were respectively 573 (170+146+133+124) and 963 (232+218+216+297), including identical + variable from the in vitro culture of both species (Figure 1). It is not clear to what these numbers “77 and 103 genes” L91 refer to. L94. The DE proteins MPB70 and MPB83 are listed as Mpt70 and Mpt83 in ref. 11 (Malone et al 2018). Please confirm they are adequately reported.

The reviewer correctly notes that Malone et al reported 573 DE genes, and 963 proteins with differential abundance, between Mbv and Mtb. However, as states in Malone et al, “Overlap of the DE lists for *M. bovis* AF2122/97 and *M. tuberculosis* H37Rv revealed 77 and 103 genes that are significantly upregulated in either species at both the RNA and the protein level, respectively”. It was these latter values that we were referring to in the manuscript. In the work described in this manuscript, we sought to triage genes and encoded proteins that may have the greatest impact on the Mbv vs Mtb in vitro phenotype. The gene *mpb70* (referred to as *mpt70* in Mtb), and the encoded protein MPB70, is among this latter group of 77 DE genes and proteins. Hence, we referred to the 77 & 103 DE lists so as to put selection of MPB70 in clearer context.

The intracellular growth was measured after 72 h of infection. Was is the doubling time of Mtb and Mbv in bacterial culture? Provide a reference. Can we expect Mtb and Mbv to divide in MDM early as 72 hrs post-infection? Mtb and Mbv were hardly treated to disperse the cells and they need to recover and adapt to MDM environment. Has been division time of Mtb or Mbv in monocyte-derived-macrophages previously reported ?

The doubling time of Mtb and Mbv in optimal culture medium is around 16 hours. There is a large number of studies (from our lab and other) showing that Mtb and Mbv replicate in macrophages in 72 hours. We can provide as example those 2 following references [8, 9].

L125-126. Cytotoxicity was reported for bMo early as 72h post-infection whereas in hMo, cytotoxicity was reported after 5 days for Mtb. How did you measure cytotoxicity, also based on based on Green Live/Dead stain?

Cytotoxicity in bMφ were based on nuclei counting, a method that we have extensively used in the lab. In contrast to hMφ, bMφ detached to the surface when they die. Green Live/Dead stain required several washes which completely clear dead cells from the well.

L161-163: This information should be part of the discussion section. That is said, autophagia is not an anti-mycobacteria pathway.

We are not sure we understand the reviewer’s comment. In the manuscript, our sentence reads: “Autophagy is an anti-mycobacterial pathway” as it has been extensively shown before (Gutierrez et al., 2004; Deretic V., 2009; Kim YS et al., 2019).

Clearance of Mtb through the regulation of autophagy is an important research topic. To confirm the activity of phagolysosomal/autophagic pathway called LC3-associated phagocytosis (LAP), the conversion of endogenous free LC3 protein (LC3-I) to its lipidated and autophagic vesicle-associated form (LC3-II) is measured (Deretic et al., 2009, Curr. Top. Microbiol. Immunol. 335:169). LC3 associates with microbe-containing phagosomes during LAP. Have you measured a change of LC3 puncta during the course of the infection with Mtb or Mbv? Results of NI and Mtb/Mbv infected Mo should be also reported as supplementary data.

We apologise, but we believe the role of LAP (non-canonical autophagy) in Mtb infection is unfortunately much more complex than this and not the main focus of the paper here. LC3B not only associates to phagosomes during LAP. Our work does not focus on the role of canonical and non-canonical autophagy during mycobacterial infections and more about the possible differential role of autophagy in bovine and human macrophages. We do not really see the point of measuring autophagy (LC3B puncta) because it has been extensively shown that different cell types and species can have different autophagic responses. Therefore comparing basal levels of autophagy, or eventually autophagy flux that provides more functional information, will not be conclusive. We agree with the reviewer that autophagic targeting was important to measure and performed these experiments and analysis considered, this is now included in **the new Figure 6**.

The formation of MNC is observed while using EVs from Mtb or Mbv supernatants. The fourth panel is about bMϕ incubated for 24 hours in serum free media. Is that correct? My understanding is that EV are precipitated in added to a complete culture media. It is not clear why the control is incubated without serum. About the other conditions? Specify in M&M section that incubation was performed in absence of serum (L519). Have you observed a difference when incubated in presence of serum? The absence of serum stimulates autophagy (serum starvation-induced autophagy in Korkmaz et al. 2013 PLoS One 8(12)). The condition of serum starvation should be clearly stated in M&M and results sections. This aspect of EVs-Mtb or EVs-Mbv-induced MNCs during serum starvation should be brought in the discussion.

We apologise for the confusion. It is correct that EVs were isolated from the supernatants of cells in culture for 24 h in serum free media. As mentioned in the Methods section, EVs are then purified and resuspended in PBS, which was used as a control. The figure was mislabelled and should be labelled as PBS.

L209. It is suggested that the nature of the inducing-MNC molecules is “proteinaceous”. Heat inactivation will unfold protein but can also affect lipid structures. Would proteinase treatment be a better control? The validation was not clearly done. This point should be reconsidered.

We consulted with our proteomics facility, and whereas is true that lipids are affected, proteinase treatment is difficult because a complex mixture of proteases will be needed and differentially affected. This is because in the paper we mention this experiment suggest the proteins are implicated.

Under normal conditions, M-CSF has been proposed as a promoter of M2 polarization due to its homeostatic expression in tissue, which induces the general M2-like phenotype of resident macrophage populations (in Trus et al. 2020 Cytokine 127: Who’s in charge here? Macrophage colony stimulating factor and granulocyte macrophage colony stimulating factor: Competing factors in macrophage polarization). Authors should discuss their choice of using the GM-CSF regulatory cytokine for primary monocyte differentiation. The use of pro-M2 Mo (M-CSF stimulated) is perhaps more appropriated as pro-M2 can polarize towards M1 or M2 and are therefore pluripotent (Jaguin et al, 2013, Cellular Immunology 281).

As explain above, inflammatory type-I macrophages are important in the TB immune response notably because they play an important role in the control of TB infection and in the lung homeostasis [1-5]. We thus used GM-CSF-derived macrophages as model of inflammatory macrophages.

This specific point is now discussed:

“Macrophage fusion is a rare event that occurs in distinct environments [51]. The first study of MNGC formation in culture showed that human monocytes could differentiate into MNGCs when exposed to certain microenvironments [52]. Since then, numerous cytokines had been identified as potent inducers of MNGCs [21, 23-25, 53]. In most experimental models using monocytes, MNGC formation occurred after several days of exposure to fusogenic factors such as IL-4, suggesting that monocytes need to mature in order to acquire fusogenic properties [25, 54]. In contrast, certain populations of primary Mϕ alternatively activated by IL-4 can induce MNGC formation relatively rapidly (i.e. in hours) when exposed to additional pro-fusogenic stimuli [21]. Thus, the fusogenic properties of Mϕ rely on multiple components, which include a specific inflammatory phenotype together with contact with other fusion partners. As MNGCs mostly originate from inflammatory Mϕ [18], we used a model of GM-CSF-derived Mϕ.”

The difference observed by electronic microscopy and confocal visualization (LAMP) between hMo and bMo (Fig. 1 F and G, respectively) is interesting. As GM-CSF promotes M1 polarization, I wonder if the use of fetal bovine serum which contains numerous cytokines/hormonal factors would influence the phenotype observed for bMo.

We did not test the impact of FBS on intracellular trafficking. However, we have ascertained that the fusogenic phenotype of Mbv-infected bMφ using a commercial medium X-Vivo (Lonza, #BEBP02-061Q), which contains an artificial serum mostly composed of human recombinant molecules, and only supplemented with GlutaMAX. Despite the absence of FBS, bMφ were still able to induce MNGC formation, which ruled out the influence of FBS in the fusogenic properties of bMφ.

GM-CSF-derived macrophages non-infected (NI) or infected with Mtb or Mbv for 24 hours. Macrophages were incubated with X-Vivo (FBS free medium) during the infection course. As shown on the graph, the number of MNGCs are significantly higher in Mbv-infected samples compared to Mtb-infected or to the non-infected control.

The methods in mammalian autophagy research have been published in Cell journal (Mizushima et al. 2010 Cell 140(3). Three principal methods are presently used to monitor the number of autophagosomes, including electron microscopy, light microscopy detection of the subcellular localization of LC3, and biochemical detection of the membrane-associated form of LC3. The light microscopy detection technique was used in current study for the identification of the subcellular localization of LC3 by immunodetection. Does the antibody recognize LC3-I or LC3-II? Does LC3B correspond to LC3-II?

The main body of methods used to monitor autophagy is in Klionsky et al., 2016 Autophagy, to which the corresponding author (MG) has contributed. LC3CB is one of the isoforms of LC3 and it is recognized by the antibody in this study. LC3-II refers to the molecule that results from the cleavage of LC3B after induction of autophagy and is commonly used to monitor autophagy.

L167. The phenomenon of multi-membrane vacuole is not documented in the literature. The description may not represent what is reported in the literature. The description should then agree or relate to the reported observations.

We disagree. The multimembrane vacuole phenotype has been extensively reported in the literature for several intracellular pathogens, including mycobacteria (Please see Figure 5 of Gutierrez et al., 2004).

The choice of the housekeeping. GAPDH has not been found stable in several study. For primary bMo, peptidylprolyl isomerise A (PPIA) was the most stably expressed gene (Taraktsoglou et al., 2011, Vet. Immunol. Immunopathol. 140(1-2)). The RALBP1-associatedEps domain-containing 1 gene (REPS1) was found by RNA-seq analysis and confirmed by QPCR to be moderately expressed in all Mbv infection conditions (Jensen et al. 2018, Infecton and Immunity. Although the endogenous GAPDH gene is stably expressed in leukocytes, it is not for bovine primary monocytes or monocyte-derived macrophages. For hMo, normalization was performed using the L34 housekeeping gene (Volpe et al., 2006 in Immunology 118:(4)).

It is recommended to test 5 housekeeping genes and to use the geometric mean the most stable ones for normalization (refer to BestKeeper in Pfaffl et al 2004 Biotechnology Letters, Determination of stable housekeeping genes, differentially regulated target genes and sample integrity: BestKeeper--Excel-based tool using pair-wise correlations).

Why normalizing with more than one gene? The group of Speleman et al. observed that a gene expression bias up to 20 fold could be created by the use of a single gene as a reference for normalization (Vandesompele et al., 2002), with consequence in the interpretation of the results when an inappropriate reference gene for qPCR data normalization is used (Dheda et al 2005 in Analytical Biochemistry 344: 141–143). It is clear that any report documenting small expression changes without validating reference genes is unacceptable as it has been shown that the use of a single non-validated reference gene results in a significant bias (ranging from more than 3-fold in 25% of the results up to 6-fold in 10% of the results). This finding is also reported in other publications, notably in Robinson et al 2007 (Veterinary Immunology and Immunopathology). The use of a single reference is therefore considered insufficient.

The reviewer raises an important point regarding choice of housekeeping genes, we did in fact assess the expression of two different endogenous genes Beta-2-Microglobulin (B2M) Glyceraldehyde 3-phosphate dehydrogenase (GAPDH) in a preliminary experiment.

Although we observed slight differences in the cycle threshold (CT) values between the conditions, these were no greater than ± 0.27 and ± 0.38 the standard deviation (SD) respectively, while target genes such as Interferon beta 1(IFNb1) showed as much as ± 1.74 SD. We concluded that either gene could be used as a normaliser. Unlike the SYBR green system used in articles cited by the reviewer, here we use a TaqMan based gene expression reporting system. Results from the two different assays can’t always be directly compared due to the inherent differences in probe design used for SYBR green based assays.

Please find below, an example of the relative mRNA expression of IFNβ in bMϕ infected with Mtb for 24 h including the normalisation with glyceraldehyde-3-phosphate dehydrogenase and beta-2-microglobulin.

Quantification by RT-qPCR of the relative fold change mRNA expression of interferon-β (IFN- β) in bMϕ infected with M. tuberculosis H37Rv (Mtb) for 24 h. Data presented are normalized to bMp infected with (Mtb). Housekeeping gene used were GAPDH (glyceraldehyde-3-phosphate dehydrogenase) or B2M (beta-2-microglobulin). bMϕ D1: donor 1; bMϕ D2: donor 2.

L283-285. The impact of M1 / M2 status and the polarization effect induced by the GM-CSF should be better developed in this section. Clearly state that GM-CSF, which supports the differentiation of monocytes into a pro-inflammatory (M1) subtype, also known as the classically activated subtype, was used for your MDM cultures. M1 pro-inflammatory status could influence the promotion of MNC formation. Indeed, the added elements L359-360 support it. The ability of MDM to generate MNCs could also be enhanced by serum deprivation therapy. These are important aspects that need to be discussed in order to guide future investigations.

In general, for human macrophages is not clear whether GM-CSF and M-CSF result in M1/M2 and we rather describe these macrophages as GM-CSF macrophages.

L291-299: very interesting!

Thank you for your enthusiasm.

The difference in subcellular localization and the presence of single or multi- membrane structures have not been discussed. The phenomenon of multi-membrane is not documented in the literature. The description may not represent what is reported in the literature. The description should then agree or relate to the reported observations. For instance, double-membrane vacuoles were monitored by electron microscopy (EM) in RAW cells (macrophage-like cells) after LPS treatment (Xu et al. 2007, Immunity 27:135). The punctate distribution of LC3, a sign of autophagosome formation, was also observed by immunofluorescence in human alveolar macrophages upon LPS stimulation (Xu et al. 2007). Is there any parallel to be made? Have you observed increased in LC3 puncta formation during the course of Mtb or Mbv infection? Electronic microscopy analysis reveals that LAP involves single-membrane structures whereas macroautophagy is expected to generate double-membrane structures surrounding cargo. A guide for the use and interpretation of autophagy is available (Klionsky et al., 2016, Autophagy 12(1):1-222. It is reported that quantification of autophagy using LC3 can be subjective (Mizushima et al. 2010). In addition, it is reported that M. tuberculosis effectively inactivates the LC3-traffinking host defenses (Köster et al. 2018, Autophagy, 14:3). How your results can be interpreted with this perspective?

The multimembrane phenotype is actually reported in the literature since 2004 (Gutierrez et al., 2004).

L334-335. Support the statement with references.

We have added two references:

“During TB inflammation, macrophage fusion occurs during granuloma progression leading to MNGCs or Langhans’ cells [17, 63].”

17.	Patterson KC, Queval CJ, Gutierrez MG. Granulomatous Inflammation in Tuberculosis and Sarcoidosis: Does the Lymphatic System Contribute to Disease? Bioessays. 2019;41(11):e1900086. doi: 10.1002/bies.201900086. PubMed PMID: 31588585.

63.	Pagan AJ, Ramakrishnan L. The Formation and Function of Granulomas. Annu Rev Immunol. 2018;36:639-65. doi: 10.1146/annurev-immunol-032712-100022. PubMed PMID: 29400999.

L94: The DE proteins MPB70 and MPB83 are listed as Mpt70 and Mpt83 in ref. 11 (Malone et al 2018). Please confirm they are adequately reported.

We assume that by “adequately reported”, the reviewer is requesting that MPB70 and MPB83 are referred to in a standard way that matches the literature. The designation MPB was introduced by Nagai et al. in 1981 [10] for proteins purified from *M. bovis* BCG, with MPT introduced by Nagai et al. subsequently in 1991, to denote proteins isolated from *M. tuberculosis* [11]. MPB70 and MPT70 are synonyms, as are MPB83/MPT83. Hence, we feel that using MPB70 in our manuscript is an adequate way to refer to this protein.

L101: add coma…demonstrate that, both in vitro and in vivo, Mbv specifically promotes…

This had been modified in the revised manuscript.

L116-117: describe the acronym RFP when first listed (red fluorescent proteins (RFP) staining of Mtb / Mbv)

The sentence now reads:” First, we optimized the infection ratios for both macrophage models by analysing the ability of hMϕ and bMϕ to phagocytose Mtb-RFP (Mtb-expressing Red Fluorescent Protein) or Mbv-RFP”

Figure 4C. The genes are missing (side of right panel: Log2 fold change Mbv/Mtb

The genes displayed in the right panel (Fig. 4C in the previous version) correspond to the same genes than Fig4. B. For more clarity both heat maps are now included in the same panel, label Fig. 4B in the revised manuscript.

Figure S1. F and G. Provide information about the condition (non-infected, incubation time, etc).

This information has been added to the legend of Figure S1.

Figure S2. Specify which panel is hMo and bMo.

hMφ and bMφ labels were added to Figure S2.

Legend of figure 1. Describe the acronym “ns” used in panel C and “NI” panel G.

This has been modified in the revised-manuscript.

Legend Figure 1: The sentence now reads: […] “(G) Quantification by RT-qPCR of the relative fold change mRNA expression of interferon-β (IFN- β) in hMϕ (left graph) and bMϕ (right graph) infected with Mtb or Mbv for 24 h. Data are normalized to Mtb. (Housekeeping gene used: GAPDH). Non-infected cells (NI) were use as control. For all the figures, p-value is considered significant when < 0.05 and indicate as follow: *p<0.05; ** p<0.01; *** p<0.001; ns: not significant.”

**References**

1.	McClean CM, Tobin DM. Macrophage form, function, and phenotype in mycobacterial infection: lessons from tuberculosis and other diseases. Pathog Dis. 2016;74(7). Epub 2016/07/13. doi: 10.1093/femspd/ftw068. PubMed PMID: 27402783; PubMed Central PMCID: PMCPMC5985490.

2.	Bryson BD, Rosebrock TR, Tafesse FG, Itoh CY, Nibasumba A, Babunovic GH, et al. Heterogeneous GM-CSF signaling in macrophages is associated with control of Mycobacterium tuberculosis. Nat Commun. 2019;10(1):2329. Epub 2019/05/28. doi: 10.1038/s41467-019-10065-8. PubMed PMID: 31133636; PubMed Central PMCID: PMCPMC6536549.

3.	Mishra A, Singh VK, Actor JK, Hunter RL, Jagannath C, Subbian S, et al. GM-CSF Dependent Differential Control of Mycobacterium tuberculosis Infection in Human and Mouse Macrophages: Is Macrophage Source of GM-CSF Critical to Tuberculosis Immunity? Front Immunol. 2020;11:1599. Epub 2020/08/15. doi: 10.3389/fimmu.2020.01599. PubMed PMID: 32793233; PubMed Central PMCID: PMCPMC7390890.

4.	Shibata Y, Berclaz PY, Chroneos ZC, Yoshida M, Whitsett JA, Trapnell BC. GM-CSF regulates alveolar macrophage differentiation and innate immunity in the lung through PU.1. Immunity. 2001;15(4):557-67. Epub 2001/10/24. doi: 10.1016/s1074-7613(01)00218-7. PubMed PMID: 11672538.

5.	Trapnell BC, Whitsett JA. Gm-CSF regulates pulmonary surfactant homeostasis and alveolar macrophage-mediated innate host defense. Annu Rev Physiol. 2002;64:775-802. Epub 2002/02/05. doi: 10.1146/annurev.physiol.64.090601.113847. PubMed PMID: 11826288.

6.	Cambier CJ, O'Leary SM, O'Sullivan MP, Keane J, Ramakrishnan L. Phenolic Glycolipid Facilitates Mycobacterial Escape from Microbicidal Tissue-Resident Macrophages. Immunity. 2017;47(3):552-65 e4. Epub 2017/08/29. doi: 10.1016/j.immuni.2017.08.003. PubMed PMID: 28844797; PubMed Central PMCID: PMCPMC5610147.

7.	Cohen SB, Gern BH, Delahaye JL, Adams KN, Plumlee CR, Winkler JK, et al. Alveolar Macrophages Provide an Early Mycobacterium tuberculosis Niche and Initiate Dissemination. Cell Host Microbe. 2018;24(3):439-46 e4. Epub 2018/08/28. doi: 10.1016/j.chom.2018.08.001. PubMed PMID: 30146391; PubMed Central PMCID: PMCPMC6152889.

8.	Jensen K, Gallagher IJ, Johnston N, Welsh M, Skuce R, Williams JL, et al. Variation in the Early Host-Pathogen Interaction of Bovine Macrophages with Divergent Mycobacterium bovis Strains in the United Kingdom. Infect Immun. 2018;86(3). Epub 2017/12/22. doi: 10.1128/IAI.00385-17. PubMed PMID: 29263113; PubMed Central PMCID: PMCPMC5820943.

9.	Lerner TR, Borel S, Greenwood DJ, Repnik U, Russell MR, Herbst S, et al. Mycobacterium tuberculosis replicates within necrotic human macrophages. J Cell Biol. 2017;216(3):583-94. Epub 2017/03/01. doi: 10.1083/jcb.201603040. PubMed PMID: 28242744; PubMed Central PMCID: PMCPMC5350509.

10.	Nagai S, Matsumoto J, Nagasuga T. Specific skin-reactive protein from culture filtrate of Mycobacterium bovis BCG. Infect Immun. 1981;31(3):1152-60. doi: 10.1128/IAI.31.3.1152-1160.1981. PubMed PMID: 7014457; PubMed Central PMCID: PMCPMC351438.

11.	Nagai S, Wiker HG, Harboe M, Kinomoto M. Isolation and partial characterization of major protein antigens in the culture fluid of Mycobacterium tuberculosis. Infect Immun. 1991;59(1):372-82. doi: 10.1128/IAI.59.1.372-382.1991. PubMed PMID: 1898899; PubMed Central PMCID: PMCPMC257751.
