# Supplementary material for: Macrophage-specific responses to human- and animal-adapted tubercle bacilli reveal pathogen and host factors driving multinucleated cell formation
Source: PLoS Pathog. 2021 Mar 15;17(3):e1009410. doi: 10.1371/journal.ppat.1009410 (PMC7993774; doi:10.1371/journal.ppat.1009410)
Supplement: Attachment — Submitted filename: Queval_point by point_REREsubmission-QC.docx [file ppat.1009410.s010.docx]

**Point by point reply to reviewers**

**Reviewer #1**

The authors have made a considerable effort to respond to my comments (and even more so to those of the other reviewers!).

I would like to make it clear that my assessment of their work as "descriptive and preliminary" was certainly not intended to downplay the importance of the quantity and quality of their work.
Now, in their responses to my comments and those of the other reviewers, the authors have convinced me that this work represents a sufficiently interesting and important body of work for the community, although in my opinion it provides almost more questions than answers, but that is the beauty of science.

The work is solid, the data is solid, its interpretation could sometimes be debated, but at least this work will have the merit of nourishing reflection in this difficult and as yet little explored field.

We are very grateful for these positive comments, which are much appreciated. We are glad that we could convince the reviewer that our work is relevant. We agree that our manuscript raises several questions, and we also believe that our data bring a novel perspective regarding the interaction of mycobacteria with their host and the specific manifestation of tuberculosis disease.

**Reviewer #2:**

*M. tuberculosis* and *M. bovis*, two closely related pathogens have interesting, poorly understood differences in host tropism. This study is aimed at understanding these differences using the each of the two pathogens in bovine and in human macrophages and assessing differences in infection phenotypes that include replication, subcellular localization, multinucleated giant cell (MNGC) formation. The problem with the work is the lack of a link of any of these findings to known differences in natural infection or to any new insights that might explain such differences. The authors have tried to link their in vitro findings of differences to the extent of MNGC formation by M. bovis versus M. tuberculosis in cows. However, even if these differences correlated with the ones they see in vitro, it is not clear how this is relevant to natural infections. After all, as the authors point out, M. tuberculosis causes MNGC formation in humans as does M. bovis in cows.

We respectfully disagree that our findings are not relevant to natural infection. As we mentioned in our manuscript, one of the major differences between *M. tuberculosis* and *M. bovis* is the differential expression of MPB70. The human adapted *M. tuberculosis* have maintained tight regulation of MPB70 expression via the SigK regulon. Conversely, *M. bovis* strains, through mutation of the RskA anti-sigma factor of SigK, express constitutively high expression MPB70 are highly successful pathogens in cattle (as well as some other animals), but generally rarely induce severe lesions in humans. *M. bovis* and *M. orygis* are responsible for cattle infection worldwide. During their evolutionary process, both strains acquired, through two distinct mutations, the ability to overexpress MPB70 through dysregulation of RskA-SigK. The fact that those two cattle-adapted strains have selected for the same phenotype suggests that MPB70 could facilitate adaptation to cattle and favour disease progression, and ultimately transmission of infection, specifically in cattle. One of our major findings is that GM-CSF derived bovine macrophages respond differently to *M. bovis* compared to *M. tuberculosis* and are more responsive to MPB70 (by forming MNGCs) than human macrophages. Although granuloma formation is well described, the immunological processes leading to granuloma formation is still not fully uncovered. The biology of macrophages is also very complex and, as discussed in our manuscript, we cannot exclude that the formation of MNGCs in human may involve other mechanism(s) or other macrophage subpopulation(s).

As another example, the sterile granulomatous disease sarcoidosis in human, leads to the formation of MNGCs similar to those observed in tuberculosis. This latter point suggests that other mechanisms, which do not require the presence of any pathogens nor MPB70, can also lead to the formation of MNGCs. We understand that our study rises many questions, but we are nonetheless convinced that our work clearly demonstrates that the interplay of host and specific bacterial factors define the outcome of infection.

In a second example of lack of link or insight from their findings, the authors show that M. tuberculosis replicates better than M. bovis in human macrophages and both replicate similarly in bovine macrophages. This might be the opposite of what one might expect, given that M. bovis is the pathogen with the broad host range. In summary, while it is clear that a great deal of work has gone into these experiments, unfortunately these findings do not add to our understanding of this interesting area of mycobacterial pathogenesis and evolution.

We respectfully disagree with the reviewer’s contention. In this work, our aim was to provide insights into the cellular mechanism that contribute to the capacity of *M. bovis* and *M. tuberculosis* to sustain in their respective host populations, an evolutionary outcome driven by repeated cycles of infection, disease and transmission. Our approach focussed on the initial cellular interactions between pathogen and host, namely the differential interaction with the macrophage. In this regard, our research revealed both contrasting pathogen replication kinetics depending on the origin of the macrophage, as well as the distinct response of the macrophage to infection in terms of MNGC formation. The cellular work is then expanded to infection of cattle with both pathogens, where *M. bovis* also showed increased MNGC formation as compared to cattle infected with *M. tuberculosis.* Our work provides insights into the natural host tropisms of *M. tuberculosis* and *M. bovis.* We are aware more work is needed in this area since studies in the bovine system are limited. We believe our work will act as the basis for further studies, by ourselves and others across the TB field, into the mechanistic basis of differential host-tropism across the MTBC.

**Reviewer #3**

The different in vivo outcome of Mtb and Mbovis has been known since the 19th century. Yet, the reason for this difference in virulence has remained elusive. In this paper, Queval and colleagues propose that Mbovis, but not Mtb, induces the formation of MNGCs, in a MPB70-dependent manner, but that this phenotype is only observed in bovine macrophages (and not human macrophages).
What makes this exciting is that it is a seminal observation that could enable the mechanistic study of virulence traits that are specific to MTBC variants. What makes this potentially confusing is that it is unclear whether it is a host- and pathogen- or a (host+pathogen)-driven phenotype. This latter question is critical for moving the field forward after this paper.

We are glad that the reviewer found our study exciting, and we share their curiosity regarding the phenotype driven by host-pathogen interactions in this context. Our study reveals an adaptive difference at the level of the macrophage-pathogen interaction between *M. tuberculosis* and *M. bovis*. Our data strongly suggests that both the pathogen (through the expression of MPB70) and the host (though exosome release following infection) participate to the formation of MNGCs by bovine macrophages. How the bacterial strains modulate the cellular response, and more specifically influence cell-to-cell communication *via* exosome delivery, is a very interesting finding. To the best of our knowledge, the correlation between pathogen, cellular exosomes and MNGC formation has not been previously reported and represents *per se* a valuable insight for moving the field forward.

However, because of the different levels of infection and the error bars, it was not entirely clear what phenotypes were specific to only a select group and what phenotypes might be more broadly observed. As examples, the only time one sees 5% MNGCs in Hmacs is with M. bovis. Many points are around zero, but can we be convinced that this only happens with Bmacs, or rather that the Bmacs are simply turned on more, such that all phenomena are more observable? The data in S4D suggests this is possible. Here, bMACs show an increase in MNGC from 4 (non infected group) to 8 with M. bovis (one star). In the same Figure, hMacs infected with M. bovis see the MNGC go from 4 (non infected) to 7 (ns); beside this, hMAcs infected with M.tb have a median of 4 MNGC (no stats shown comparing M. bovis to Mtb).

We understand the concern of the reviewer. In our manuscript, we mostly focus on the characterisation of MNGCs formed from bovine macrophages (bMacs). Although it is true that we can detect a basal level of so called “MNGCs” in hMacs, they are morphologically very different compared to the those observed in Mbv-infected bMacs. Indeed, we observed that MNGCs derived from hMacs were mostly bi-nucleated cells without any increase of cell size. In light of the constructive comment of the reviewer, we realized that this difference was not sufficiently emphasized in our manuscript and could be confusing to the readers. For more clarity, we have included a comparative analysis of the MNGCs induced by Mbv in both bovine and human macrophages (**new Figure S3**).

In the new figure S3: (**C**) GM-CSF derived bovine (bMϕ) or human macrophages (hMϕ) were infected with Mbv-RFP (red) for 24 h. PFA-fixed infected cells were stained with phalloidin-Alexa Fluor 488 (actin, green). DAPI (blue) was used to stain nuclei. Images presented above were acquired using a confocal microscope. Images were analysed using Harmony software (PerkinElmer). Actin stain was used to mask the cell bodies and determine the number and the area of the cells detected. DAPI staining was used to segment and count the number of nuclei in each cell. Cells containing 2 or more nuclei were considered. For each cell represented by a grey dot, values were plotted as nuclei number as a function of the cell’s area (µm^2^). Scale bar, 50 µm.

These data had been included in the manuscript, Results section page 7:

*“Multinucleation of bMϕ occurred only with live bacteria since paraformaldehyde-killed-Mbv (PFA-Mbv) showed similar numbers of MNGCs to both Mtb-infected and uninfected Mϕ (Fig. S3A and S3B). This effect was restricted to bMϕ, since MNGCs were almost absent in hMϕ infected either with Mtb or Mbv (Fig. 2C). Importantly, Mbv-infected bMϕ presented a wide range of multinucleated cells going up to over 10 nuclei associated with an increase of the cell size (Fig S3?). In contrast, the few multinucleated hMϕ detected were mostly bi-nucleated with a small variation of their size (Fig S3C). At the ultrastructural level, we confirmed that the multiple nuclei were contained in a single cell (Fig. 2D and* *Fig. S3C).”*

I can envision two key experiments to help clarify this issue and resolve whether this phenotype truly depends on the host-pathogen mix and a third experiment that could potentially lock down the role of MPB70.

1) In 3F, if the phenotype requires bMac EVs, can the authors prepare bMac EVs in a pathogen-independent manner, e.g. after phagocytosis of latex beads, to show whether the host cell on its own can produce the MNGC phenotype?

An extracellular vesicle (EV) profile was consistently detected in our proteomic studies (Figure 4) in all the conditions tested, including uninfected cells. This suggests there is a basal production of EVs in the bMac. The proposed experiments with latex beads are interesting, however, we would like to emphasize that because *M. tuberculosis* does not induce MNGCs formation, it represents the most appropriate control in this experiment, and support that MNGCs formation is not only due to phagocytosis.

2) In 3F, to test whether the M. bovis pathogen on its own can result in MNGC formation, can the authors simply prepare a culture filtrate and determine whether the culture filtrate, on its own, results in MBNGC formation?

3) As a modification on this second experiment, can the authors prepare a culture filtrate of M. bovis disrupted for MPB70 to determine whether a bacterial-induced MNGC phenotype is diminished when MPB70 is absent?

This is a very good point. By using bacterial culture filtrates, the concentration of various bacterial factors including lipids or secreted virulence factors that affect the activation state of the macrophages will be very high, affecting the interpretation of the results. In order to address the reviewer concern regarding the role of MPB70 and whether the phenotype depends on the host-pathogen combination, we generated an Mtb-H37Rv strain overexpressing MPT70 (MPT70 is the homologous of MPB70 but present in the human-adapted *M. tuberculosis* strains). Interestingly, we found that the expression of MPT70 by Mtb did not rescue the MNGCs formation observed with Mbv. This provide evidence that MPB70 is necessary for MNGC formation in bMac only in the context of Mbv infection (**new Figure 3**).

*M. bovis* and *M. orygis* are mycobacteria responsible for cattle infection worldwide. During their evolutionary process, both strains acquired, through two distinct mutations, the ability to overexpress MPB70. (please see our answer to the reviewer 2). This latter point suggests that MPB70 could facilitate adaptation, disease progression, and ultimately transmission of infection, specifically in cattle. The fact that MPB70 contributes to MNGC formation, especially in Mbv context, could explain why the most pathogenic mycobacteria for cattle have during their evolution selected mutation leading to high level of MPB70 production.

Finally, the specific role of MPB70 in Mbv infection context together with pathogen-specific nature of EVs released by the bMac after Mbv infection support that the MNGC formation results from a mix between the specific host response and the pathogen.

We have added this new panels in the Figure 3:

Legend Figure 3:

*(D) Fluorescence confocal images of bMϕ infected with Mbv WT, Mtb, Mtb-MPB70+ for 24 h. Uninfected cells had been used as a negative control. Cell nuclei are stained with DAPI (cyan) and bacteria-RFP and actin are visualized in red and grey, respectively. Yellow arrows point the multi-nucleated cells. Scale bar, 50 µm (E) Quantification of MNGCs from each experiment displayed in D. Graph represents the quantification of MNGCs for each condition tested where each dot represents one bovine donor. Data shown are representative of two independent experiments.*

and include the description and data interpretation in the results section page 8:

“*MPT70 (homologous to MPB70 in human-adapted strains) is not completely absent but expressed at a very low level by Mtb strains [11, 14]. We then wanted to examine whether MPB70/MPT70 itself control MNGC formation or whether other factors were required for bMϕ multinucleation. We thus generate an Mtb strain constitutively expressing MPT70 (Mtb-MPT70+; Figure S5E and S5F). Next, we infected bMϕ with Mbv WT, Mtb WT or Mtb-MPT70+ for 24 h, and analysed the numbers of MNGCs (Fig. 3D and 3E). As observed before, Mbv WT induced MNGCs. the number of MNGCs was significantly increased in bMϕ. Interestingly, the number of MNGCs in bMϕ infected Mtb-MPT70+ remained similar to the one counted in both uninfected or Mtb WT infected cells (Fig. 3D and 3E). This suggests that, unlike Mtb, Mbv induces a specific bMϕ response, which together with MPB70 expression enable the formation of MNGCs.*”

We also added in the new Figure S5 the description of the plasmid and the western blot showing the secretion of MPB70.

Legend Figure S5:

*“(E) Graphical map of the plasmid pGM221-1 used to generate the strain Mtb overexpressing MPT70 (Mtb-MPT70+). The Mtb-H37Rv gene mpt70 is expressed under the control of hsp60 promotor. The clonality of the Mtb-MPT70+ starin was sustained by the Kanamycin resistance cassette carried by the plasmid pGM221-1. (F) Western immunoblot for detection of MPB70 and its homologous MPT70 in the supernatant of the Mbv WT, Δmpb70, Mbv-Compl, Mtb WT and Mtb-MPT70+ strains. A 23 kDa band corresponding to MPB70 was detected for Mbv WT, Mbv-Compl and MTB-MPT70+ strains but not for Mbv Δmpb70 and Mtb WT strains.”*

I don’t understand why the Y-axis is jumping around. IN 2B, the BMacs go up to 15 but the HMacs go up to 20. Since the results are lower with the Hmacs, why is the axis scaled to a higher number? In 4F it goes to 8. In S3B it goes up to 40.

As requested, the y-axis in graph Fig. 2B (bMac) has been increased to 20. We would like to emphasize that those experiments have been done over an extended period of time with different cows. It is true that, as with other models, bMac are subject to donor-to-donor variations, which can result from the intrinsic immune status of the cow (due to different points in the lactation cycle, hormonal balance, the time of the year when the experiment was performed, etc). For Figure S3B, the scale bar difference is due to the variation in the response between the cows tested. We aimed here to be transparent and not remove any outliners (low or high values) in order to present the data directly as they appeared to us.

There are times when the bMac data is presented before the hMac, and times it goes the other way around. I propose it will be more readable if there is a standard convention. This also applies to the writing, where some sentences presented the data in reverse order for variety, but it can lead to confusion.

We thank the reviewer for pointing this out. During the preparation of this manuscript, several options were considered, and figures and text were organised to reflect the argumentative structure.

For example, in the first part of the manuscript we choose to discuss hMac first because they are a well described model for mycobacteria intracellular trafficking compare to bMac. In the second part of the manuscript, the bMac appeared first because they presented the phenotype of MNGCs; while hMac are mainly used as control. We have done our best, with the recommendation of all the reviewers, to clearly specify in main text, figures and legends the cell type. We do think that changing the order at this stage will affect the whole organisation of the manuscript and the argumentative flow.
